# Hymeneal

**Published:** 1875-06

**Authors:** 


### *Hymeneal.*

Married, in Navasota, Texas, on the 15th of April, by Rev. J. M. Weston, Mr. Ben. B. Baxter, jr., of Rockdale, Milam county, to Miss Elizabeth, youngest daughter of Dr. A. R. Kilpatrick, one of the Assistant Editors of the SOUTHERN MEDICAL RECORD.

